# A long noncoding RNA GTF2IRD2P1 suppresses cell proliferation in bladder cancer by inhibiting the Wnt/β‑catenin signaling pathway

**DOI:** 10.7717/peerj.13220

**Published:** 2022-04-11

**Authors:** Zhuo Huang, Hongbin Gao, Liangliang Qing, Biao Wang, Chaoyong He, Ning Luo, Chuncheng Lu, Shipeng Fan, Peng Gu, Hui Zhao

**Affiliations:** 1 Department of Urology, First Affiliated Hospital of Kunming Medical University, Kunming Medical College, Kunming, Yunnan, People’s Republic of China; 2 Clinical Research Center for Chronic Kidney Disease, First Affiliated Hospital of Kunming Medical University, Kunming Medical College, Kunming, Yunnan, People’s Republic of China

**Keywords:** Bladder cancer, Long noncoding RNA, Cell cycle, Proliferation, Signaling pathway

## Abstract

**Background:**

There is growing evidence that long non-coding RNAs (LncRNAs) are key in the development of a variety of human tumors. However, the role of lncRNA GTF2IRD2P1 has not been well studied in cancer. The impact of GTF2IRD2P1 on the biological function and clinical relevance in bladder cancer is largely unknown. This study aimed to investigate the biological role of GTF2IRD2P1 in bladder evolution and carcinogenesis.

**Methods:**

We used bioinformatics to obtain the lncRNA GTF2IRD2P1 from bladder urothelial carcinoma (BLCA) in The Cancer Genome Atlas (TCGA) database. The expression of lncRNA GTF2IRD2P1 was detected by qRT-PCR. The CCK8 assay and flow cytometry were used to detect the lncRNA GTF2IRD2P1 function on the proliferation of bladder cancer cells. A western blot was used to calculate the protein level of cell cycle proteins and Wnt signaling pathway proteins. The effect of lncRNA GTF2IRD2P1 on tumorigenesis of bladder cancer was confirmed by a xenograft nude mouse model.

**Results:**

GTF2IRD2P1 expression was found to be lower in both human bladder cancer tissues and cell lines (UM-UC-3, RT4, and 5637), and elevated in T24 compared to the corresponding normal controls. GTF2IRD2P1 expression was also enhanced after transfection of UM-UC-3 cells with the overexpression vector. Meanwhile, overexpression of GTF2IRD2P1 inhibited the proliferation of UM-UC-3 and prolonged the cell cycle. The silencing of GTF2IRD2P1 significantly increased the proliferation and shortened the cell cycle of T24 cells and induced Wnt signaling activity to promote the progression of bladder cancer. Similarly, the transplanted tumor nude mouse model demonstrated that silencing GTF2IRD2P1 strengthens the progression of bladder cancer by targeting the Wnt signaling pathway.

## Introduction

Bladder cancer (BCa) is among the ten most common types of cancer in the world, the fourth most common malignancy in men, and one of the most common malignancies in women (Richters, Aben & Kiemeney, 2020; Lenis et al., 2020). Globally, there are approximately 550,000 new cases each year, of which roughly 200,000 patients die (Bray et al., 2018). Despite significant advances in clinical therapies such as surgery, chemotherapy, and radiation therapy over the past few decades, the 5-year survival rate for BCa patients remains low (Humphrey et al., 2016; Alfred Witjes et al., 2017; Babjuk, 2017; Nadal & Bellmunt, 2019; Knowles & Hurst, 2015). The lack of early diagnosis and effective treatment may result in a poor clinical prognosis for BCa patients (Carneiro et al., 2015). Therefore, the discovery of novel BCa target molecules is of great clinical importance for the treatment of BCa.

Long non-coding RNAs (lncRNAs) are defined as non-coding RNAs with at least 200 nucleotides that do not encode proteins (Bunch, 2018). LncRNA plays an important role in regulating the multiple physiological activities of cells (Xie et al., 2017). Many studies have shown the abnormal expression of lncRNAs in tumors, and thus they are potential therapeutic targets for some cancers (Liang et al., 2020; Zha et al., 2021; Long et al., 2021; Li et al., 2020). LncRNA may be used for therapeutic targeting and as a reliable diagnostic and predictive factor for the survival and tumor recurrence in patients with bladder tumors (Quan et al., 2018; He et al., 2019). The occurrence and progression of malignant tumors can be regulated by lncRNAs in a variety of ways. For example, the competitive adsorption of miR-132 by lncRNA SNHG5 through sponge function, which regulates SOX4 expression, contributes to the development of cervical cancer (Zhang et al., 2021). The lncRNA DNAJC3-AS1 regulates fatty acid synthase through the EGFR pathway, thereby promoting colorectal cancer progression (Tang et al., 2020). LncRNA PCAT1 can interact with DKC1 to activate the VEGF/AKT/Bcl-2/caspase9 pathway, thereby regulating non-small cell lung cancer (NSCLC) cell proliferation, invasion, and apoptosis (Liu et al., 2021). A variety of lncRNAs have been reported to play critical role in BCa biology, including lncRNA MAFG-AS1, lncRNA SLC16A1-AS1 and lncRNA SNHG1 (Tang et al., 2021; Logotheti et al., 2020; Xu et al., 2020). However, the role of lncRNAs in BCa are relatively unknown, specifically that of lncRNA GTF2IRD2P1.

We obtained the lncRNA GTF2IRD2P1, which is one of the most significantly expressed lncRNAs in BCa samples, using bioinformatic analysis of The Cancer Genome Atlas (TCGA) database. We then used survival analysis to determine that GTF2IRD2P1 was a highly expressed lncRNA with a very significant protective effect on the overall survival (OS) of BCa patients. Finally, we used Gene Set Enrichment Analysis (GSEA) to predict the possible involvement of GTF2IRD2P1 in the Wnt signaling pathway. We validated our prediction by exploring the role of lncRNA GTF2IRD2P1 in BCa cells and the downstream Wnt pathway by performing gain and loss of function of lncRNA GTF2IRD2P1. Our findings indicate that elevated lncRNA GTF2IRD2P1 significantly inhibited Wnt signaling. The results of this study suggest that lncRNA GTF2IRD2P1 downregulation promotes BCa development through activation of the Wnt signaling pathway.

## Materials and Methods

### Bioinformatics analysis based on TCGA BLCA dataset

We downloaded the RNA-seq expression matrix and clinical data of TCGA-BLCA from UCSC’s Xena database (https://xenabrowser.net/datapages/) to screen for lncRNAs that are differentially expressed in BCa patients using the detection platform polyA+ IlluminaHiSeq. The TCGA-BLCA cohort was comprised of 426 cases (19 cases of normal bladder tissue, 407 cases with tumor tissue of BCa origin). Additional details may be found from the portal. We then analyzed the expression correlation of 20,530 genes in the TCGA-BLCA expression matrix with GTF2IRD2P1 by using the psych package in R language with the statistical method Pearson correlation and the Pearson product-moment correlation coefficient. GSEA analysis was performed using the cluster profile package, and the gene set data was downloaded from misgdb using the cancer gene set (https://www.gsea-msigdb.org/gsea/msigdb/index.jsp). Thus, we created the hallmark feature gene set (h. all. V 7.2. Entrez. gmt).

### Clinical sample

We acquired thirty BCa tumor tissue samples with the corresponding adjacent normal tissue (5 cm from the edge of the cancerous tissue) from patients undergoing surgery at the First Affiliated Hospital of Kunming Medical University between October 2018 and June 2021. Written informed consent was obtained from each patient according to committee policy. A diagnosis of uroepithelial carcinoma was independently confirmed by two pathologists in all specimens. Ethical consent was approved by the Human Research Ethics Review Committee of the First Affiliated Hospital of Kunming Medical University (approval number: (2021) Lunshen L No. 33).

### Extraction of RNA and the qRT-PCR

The extraction of the total RNA from BCa tissues and cell lines was performed using a TRIzol kit (Thermo Fisher Scientific, Waltham, MA, USA), followed by cDNA synthesis using the Reverse Transcription Kit (GeneCopoeia, Guangzhou, China). Quantitative real-time polymerase chain reaction (qRT-PCR) was conducted using BeyoFast™ SYBR Green qPCR Mix (Bio-Rad, Shanghai, China) based on the manufacturer’s guidelines. The primer sequences were as follows: 5′-GGATACGGTGCCCACGA-3′, 3′-TTTCAAGACATAGCTGACCAGCTT-5′. GAPDH: 5′-ACAGCCTCAAGATCATCAGC-3′, 3′-TAGCACCTTCCTGAGTACTGG-5′. GAPDH was applied as an internal control for gene expression standardization. All reactions were performed in triplicate and relative gene expression was calculated using the 2^−ΔΔCt^ method.

### Western blotting

We extracted total proteins from cells, animal tissues and human tissue specimens, respectively, and determined protein concentrations using a BCA kit (Beyoncé, Shanghai, China). Total proteins were solubilized using RIPA lysis buffer (Beyoncé, Shanghai, China) containing various protease inhibitors. The total protein was separated by SDS-PAGE and was transferred to PVDF membranes (Millipore, Burlington, MA, USA) by electroblotting. The membranes were blocked with 5% skimmed milk for 1 h at room temperature. The membranes were incubated overnight at 4 °C with specific primary antibodies. The membrane then was incubated with the secondary antibody at room temperature for 2 h and was detected with an ultrasensitive ECL chemiluminescence kit (New Saimei, Suzhou, China) and imaged with a chemiluminescence imaging system (Shanghai Tennant, Shanghai, China). The primary antibodies were as follows: Cyclin A2 (#18202-1-AP; Proteintech, Wuhan, China), Cyclin D1 (#60186-1-Ig; Proteintech, Wuhan, China), β-catenin (#51067-2-AP; Proteintech, Wuhan, China), TCF4 (#GR3295722-2; Proteintech, Kunming, China), E-cadherin (#20874-1-AP; Proteintech, Wuhan, China), Anti-mouse IgG (H+L) (#14709; CST, Parsons, KS, USA), Anti-rabbit IgG (H+L) (#14708; CST, Parsons, KS, USA), GAPDH mouse-derived (#60004-1-Ig; Proteintech, Wuhan, China), and GAPDH rabbit source (#10494-1-AP; Proteintech, Wuhan, China).

### Cell culture

We purchased four BCa cell lines (5637, RT4, T24 and UM-UC-3) and the standard bladder cell line SV-HUC-1 from the American Typical Culture Collection (ATCC). All were simultaneously cultured in RPMI-1640 medium and F-12K medium with 10% fetal bovine serum (FBS) and 1% dual antibody (P/S) as recommended by the manufacturer. These cells were then cultured in a humidified incubator at 37 °C with 5% CO_2_.

### Lentiviral packaging and vector construction

The pLVX-puro-GTF2IRD2P1 group and the lentivirus negative control group (pLVX-puro) were determined to be the overexpression groups; the pLVX-shGTF2IRD2P1-1-puro group, the pLVX- shGTF2IRD2P1-2-puro group, and the lentivirus negative control group (pLVX-shRNA2-puro) were the low expression groups and were purchased from Acura Biotechnology (Hunan, China). We synthesized the GTF2IRD2P1 sequence and subcloned it into plasmids pLVX-IRES-ZsGreen1 and pLVX-shRNA2-puro to obtain the overexpression and low expression group plasmids, respectively. Each plasmid was then packaged with lentivirus. Transfection was performed with the Liposome 2000 reagent (ThermoFisher Scientific, Waltham, MA, USA) based on the manufacturer’s protocol.

### The cell growth test

The assay for cell proliferation was determined using the Cell Counting Kit 8 (CCK-8). Cells were inoculated into 96-well plates with 5 × 10^^3^ cells per well in triplicate. Ten microliters of CCK-8 reagent were added to each well at 0, 24, 48, and 72 h and incubated in a CO_2_ incubator for 2 h. The absorbance was measured at 450 nm. All experiments were repeated at least three times.

### Analysis of the cell cycle

The resulting cells were digested with pancreatic protease and immobilized in 75% ethanol at 4 °C for up to 24 h. The supernatant was discarded by centrifugation and incubated for 1 h with PI staining solution containing RNAase A. The cycle of the cells was subsequently detected by flow cytometry (Eisen, Lansing, MI, USA). The experiment was repeated three times.

### Nude mice tumorigenesis assay

A total of 12 BALB/c nude mice, between 5 to 6 weeks old, were provided by Hunan Sleek Jingda Laboratory Animal Co. (Changsha, China). The mice were divided into two groups. In the LV-NC groups, T24 cells (5 × 10^^6^) infected with lentivirus were subcutaneously inoculated into the armpits of mice as a negative control group (*n* = 6). In the LV-shRNA group, T24 cells infected with Lv-shGTF2IRD2P1-2 were inoculated into remaining mice using the same procedure. The volume (V) of the tumor was obtained as follows: V = (ab^2^)/2, with “a” indicating the long diameter of the tumor and “b” indicating the short diameter of the tumor. All experiments concerning animals were carried out following the guidelines for animal experiments approved by the Laboratory Animal Ethics Committee of Kunming Medical University.

### An analysis of statistics

Statistical analysis was performed using SPSS 23.0 and GraphPad Prism 8 software. Survival curves were calculated using the Kaplan-Meier method and p-values were calculated using the log-rank test. Two independent groups were analyzed using the t-test if the data conformed to a normal distribution and the Mann-Whitney rank-sum test or the Kolmogorov-Smirnov test if the data did not conform to a normal distribution. All data were expressed as mean ± SEM or SD, and *p* < 0.05 was considered statistically significant.

## Results

### Identification of key BCa lncRNAs based on TCGA and analysis of pathway enrichment based on the KEGG and GSEA

**Figure 1:**
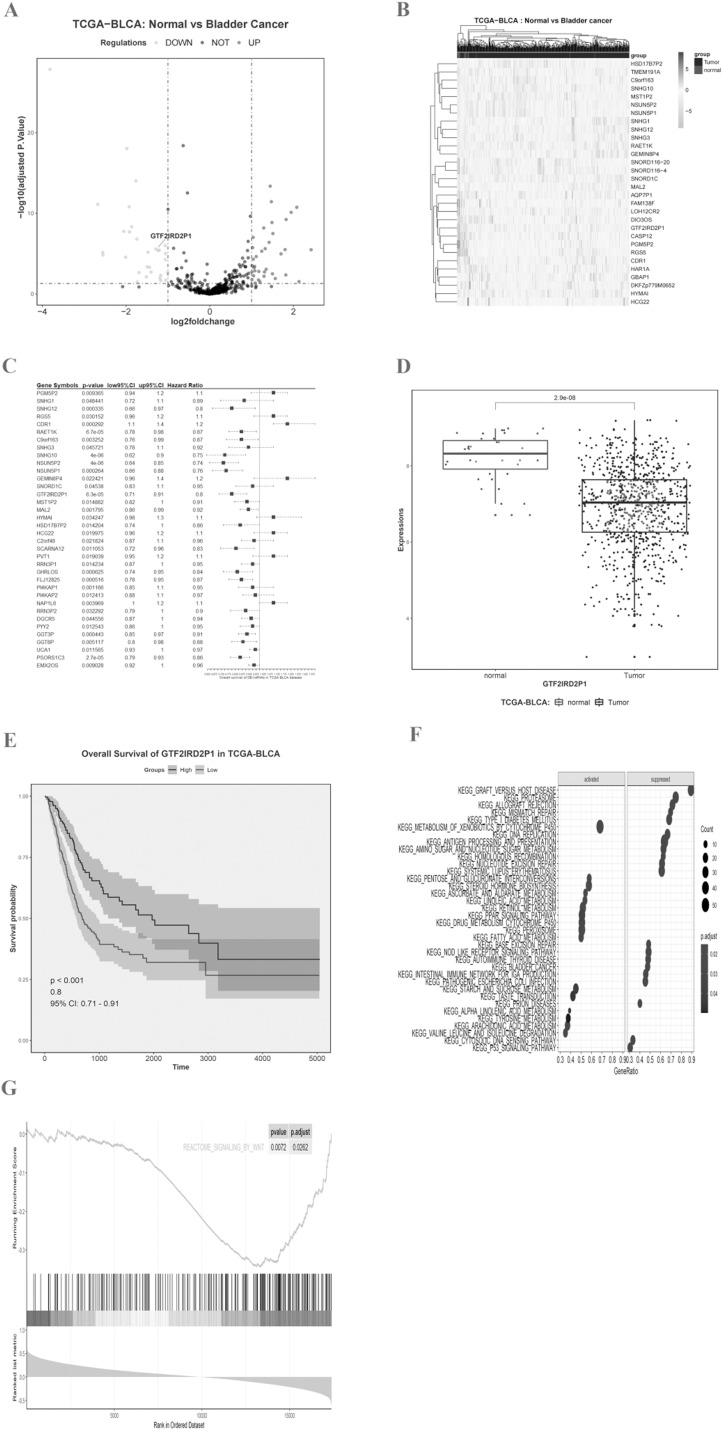
Identification of key BCa lncRNAs based on the TCGA and its analysis of pathway enrichment based on the KEGG and GSEA. (A and B) Volcano plot and heat plot differentially demonstrate the distribution of lncRNAs. (C) Cox regression analysis of single factors for differentially expressed lncRNAs. (D) Analysis of differential expression of GTF2IRD2P1 in BCa tissues and normal tissues in the TCGA database using box line plots. (E) Survival analysis of GTF2IRD2P1 in the TCGA database. (F and G) Bioinformatics predicted signaling pathways significantly associated with GTF2IRD2P1.BCa, bladder cancer; lncRNAs, long noncoding RNAs.

We imported the TCGA-BLCA expression matrix into R and analyzed it using the limma toolkit to obtain 30 lncRNAs that were significantly up-regulated (logFC > 1, *p*.adj < 0.05) and 36 lncRNAs that were significantly down-regulated (logFC < −1, *p*.adj < 0.05). The volcano (Fig. 1A) and heat map (Fig. 1B) differences show the distribution of lncRNA. One-way Cox regression analysis of the 66 lncRNAs was then performed using R survival and survminer packages with a set minimum threshold between the two sets of cutoffs at 0.2. The accompanying table of clinical survival characteristics was downloaded from the Xena database at UCSC (https://xenabrowser.net/datapages/). We used the overall survival time (OS.time) data, as well as OS survival event data to correlate with the expression of relevant lncRNAs. Ultimately 36 of 66 lncRNAs were associated with overall survival in BLCA patients (Fig. 1C), and the five most statistically significant (*p* < 0.0001) lncRNAs were SNHG10, NSUN5P2, PSORS1C3, GTF2IRD2P1, and RAET1K (Table 1). GTF2IRD2P1 was found to be one of the most significant molecules associated with overall survival in BCa. Its co-expressed genes were able to significantly negatively enrich the BCa pathway of the Kyoto Encyclopedia of Genes and Genomes (KEGG) (*p*.adjust < 0.05, enrichment score = −0.503) (Fig. 1F). Box plot analysis showed that the expression level of GTF2IRD2P1 was significantly lower in tumor tissues than in normal tissues adjacent to cancerous tissues (Fig. 1D). In addition, low-GTF2IRD2P1 expressing patients had a significantly worse prognosis than high-expressing patients according to Kaplan Meier analysis (Hazard Ratio < 1, *p* < 0.001) (Fig. 1E). Lastly, we used GSEA to predict the possible involvement of GTF2IRD2P1 in the Wnt signaling pathway (Fig. 1G).

**Table 1:** 36 lncRNAs differentially expressed in BCa that was also associated with survival (one-way Cox regression analysis).

Gene symbol	*p*.val	Hazard ratio	95% CI
SNHG10	3.62E−06	Hazard Ratio = 0.75	95% CI [0.62–0.9]
NSUN5P2	4.11E−06	Hazard Ratio = 0.74	95% CI [0.64–0.85]
PSORS1C3	2.75E−05	Hazard Ratio = 0.86	95% CI [0.79–0.93]
GTF2IRD2P1	6.33E−05	Hazard Ratio = 0.8	95% CI [0.71–0.91]
RAET1K	6.70E−05	Hazard Ratio = 0.87	95% CI [0.78–0.98]
NSUN5P1	0.000264	Hazard Ratio = 0.76	95% CI [0.66–0.88]
CDR1	0.000292	Hazard Ratio = 1.2	95% CI [1.1–1.4]
SNHG12	0.000335	Hazard Ratio = 0.8	95% CI [0.66–0.97]
GGT3P	0.000443	Hazard Ratio = 0.91	95% CI [0.85–0.97]
FLJ12825	0.000516	Hazard Ratio = 0.87	95% CI [0.78– 0.95]
GHRLOS	0.000625	Hazard Ratio = 0.84	95% CI [0.74–0.95]
PI4KAP1	0.001166	Hazard Ratio = 0.95	95% CI [0.85–1.1]
MAL2	0.001795	Hazard Ratio = 0.92	95% CI [0.86–0.99]
C9orf163	0.003252	Hazard Ratio = 0.87	95% CI [0.76–0.99]
NAP1L6	0.003969	Hazard Ratio = 1.1	95% CI [1–1.2]
GGT8P	0.005117	Hazard Ratio = 0.88	95% CI [0.8–0.98]
EMX2OS	0.009028	Hazard Ratio = 0.96	95% CI [0.92–1]
PGM5P2	0.009365	Hazard Ratio = 1.1	95% CI [0.94–1.2]
SCARNA12	0.011053	Hazard Ratio = 0.83	95% CI [0.72–0.96]
UCA1	0.011565	Hazard Ratio = 0.97	95% CI [0.93–1]
PI4KAP2	0.012413	Hazard Ratio = 0.97	95% CI [0.88–1.1]
PYY2	0.012543	Hazard Ratio = 0.95	95% CI [0.86–1]
HSD17B7P2	0.014204	Hazard Ratio = 0.86	95% CI [0.74–1]
RRN3P1	0.014234	Hazard Ratio = 0.95	95% CI [0.87–1]
MST1P2	0.014882	Hazard Ratio = 0.91	95% CI [0.82–1]
PVT1	0.019039	Hazard Ratio = 1.1	95% CI [0.95–1.2]
HCG22	0.019975	Hazard Ratio = 1.1	95% CI [0.96–1.2]
C2orf48	0.021824	Hazard Ratio = 0.96	95% CI [0.87–1.1]
GEMIN8P4	0.022421	Hazard Ratio = 1.2	95% CI [0.96–1.4]
RGS5	0.030152	Hazard Ratio = 1.1	95% CI [0.96–1.2]
RRN3P2	0.032292	Hazard Ratio = 0.9	95% CI [0.79–1]
HYMAI	0.034247	Hazard Ratio = 1.1	95% CI [0.98–1.3]
DGCR5	0.044556	Hazard Ratio = 0.94	95% CI [0.87–1]
SNORD1C	0.04538	Hazard Ratio = 0.95	95% CI [0.83–1.1]
SNHG3	0.045721	Hazard Ratio = 0.92	95% CI [0.78–1.1]
SNHG1	0.048441	Hazard Ratio = 0.89	95% CI [0.72–1.1]

**Notes:**

A total of 36 of 66 lncRNAs were associated with overall survival in BCa patients, and the five most statistically significant (*p* < 0.0001) lncRNAs were SNHG10, NSUN5P2, PSORS1C3, GTF2IRD2P1, and RAET1K.

BCa, bladder cancer; lncRNAs, long noncoding RNAs.

### Relationship of GTF2IRD2P1 with the clinicopathological features of BCa patients

BCa patients were divided into low-and high-GTF2IRD2P1 expression groups according to the median expression value of GTF2IRD2P1 (0.47). The findings from this analysis, summarized in Table 2, showed that GTF2IRD2P1 expression was associated with tumor grading (*p* = 0.01). In contrast, no correlation was found between GTF2IRD2P1 expression and other features, including age, gender, smoking, tumor size and lymph node metastasis (all *p* > 0.05).

**Table 2:** Relationship of GTF2IRD2P1 with the clinicopathological features of BCa patients.

Features	Total no. *n* = 30	GTF2IRD2P1 expression		*p* values
		Low (*n* = 15)	High (*n* = 15)	
Age (years)				0.372
≤65	16	8	8	
>65	14	7	7	
Gender				0.999
Female	6	2	4	
Male	24	13	11	
Smoking				0.999
No	10	4	6	
Yes	20	11	9	
Tumor size (cm)				0.669
≤3	21	11	10	
>3	9	4	5	
Lymph node metastasis				0.999
No	28	13	15	
Yes	2	2	0	
Tumor grading				0.010
Low	9	1	8	
High	21	14	7	

**Notes:**

GTF2IRD2P1 expression was associated with tumor grading (*p* = 0.01). However, no correlation was found between GTF2IRD2P1 expression and other features, including age, gender, smoking, tumor size, and lymph node metastasis (all *p* > 0.05).

BCa, bladder cancer.

### GTF2IRD2P1 is downregulated in BCa tissues and expressed in cell lines

**Figure 2:**
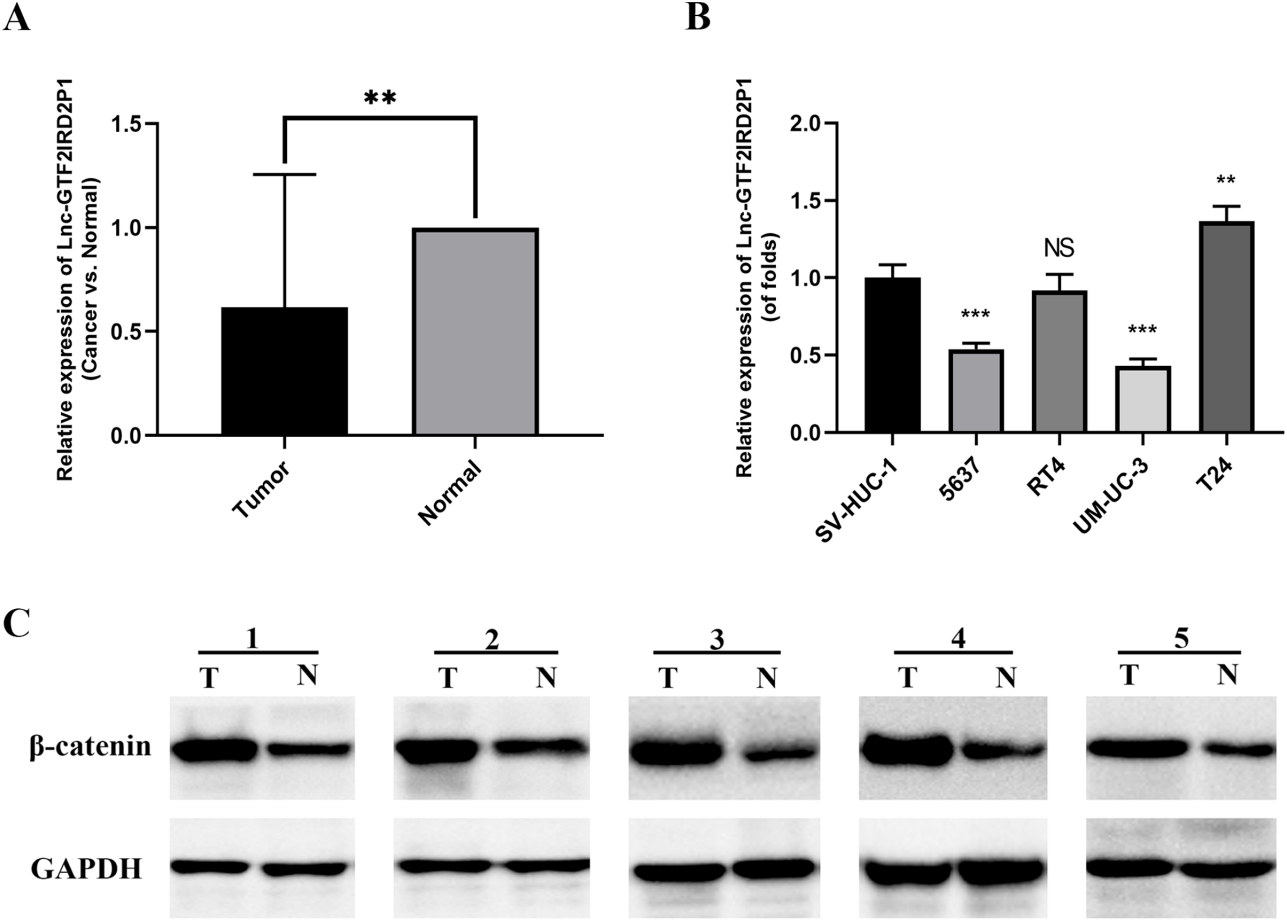
Expression of GTF2IRD2P1 and Wnt pathway proteins in BCa tissues. (A) Expression levels of GTF2IRD2P1 in patients with BCa. (B) Expression levels of GTF2IRD2P1 in four types of BCa cell lines and the standard bladder cell line SV-HUC-1. (C) Expression levels of β-catenin protein in five patients with BCa Not significant (NS), p** < 0.01, p*** < 0.001. BCa, bladder cancer.

We measured the expression of GTF2IRD2P1 in cancer and paraneoplastic tissues from 30 BCa patients using by RT-qPCR to further validate our findings. The results showed that the expression of GTF2IRD2P1 in BCa tissues was significantly lower than that in normal tissues adjacent to cancer (Fig. 2A). A significant downregulation of GTF2IRD2P1 expression in neoplastic tissues was observed compared to the neighboring normal tissues. Therefore, we used RT-qPCR to validate the expression levels of GTF2IRD2P1 in the standard bladder cell line SV-HUC-1 and BCa cell lines standardized by GAPDH. Four BCa cell lines (5637, RT4, UM-UC-3 and T24) had different levels of GTF2IRD2P1 expression compared to SV-HUC-1. GTF2IRD2P1 had a significantly lower expression in BCa cell lines (5637 and UM-UC-3) *vs* SV-HUC-1, while the opposite was true for the BCa cell line T24 (Fig. 2B). In addition, protein expression of β-catenin, a key protein in the Wnt signaling pathway, was significantly upregulated in BCa tissues compared to the corresponding adjacent normal bladder tissues (Fig. 2C).

### Effect and role of GTF2IRD2P1 in cell proliferation and cell cycle by *in vitro* experiments

**Figure 3:**
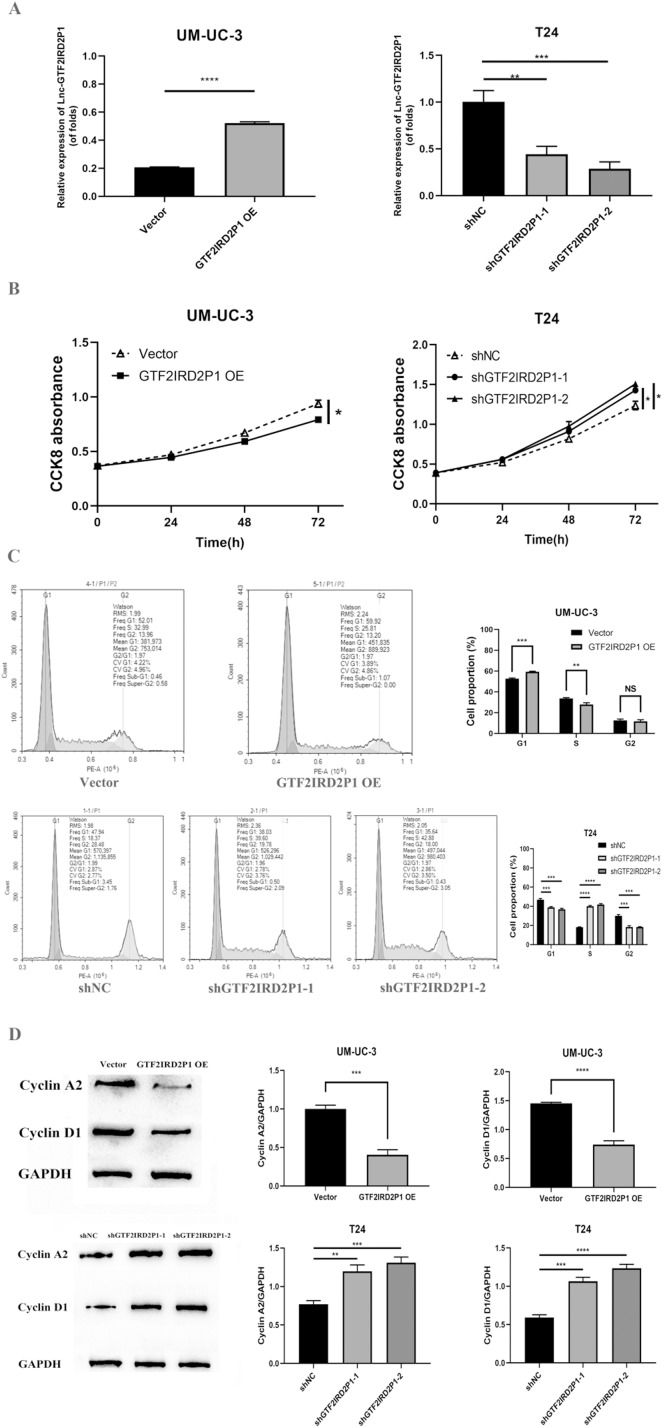
Effect of GTF2IRD2P1 on proliferation and cell cycle of BCa cells by *in vitro* assay. (A) Validation of the expression efficiency of GTF2IRD2P1. (B) CCK8 assay to detect the influence of GTF2IRD2P1 on the proliferation of BCa cells. (C) Flow cytometric analysis of the impact of GTF2IRD2P1 on BCa cell cycle. (D) Western blot to detect the expression of cell cycle proteins. Not significant (NS), p* < 0.05, p** < 0.01, p*** < 0.001, p**** < 0.0001. BCa, bladder cancer.

In UM-UC-3 and T24 cells, the effect of GTF2IRD2P1 overexpression (GTF2IRD2P1 OE) and GTF2IRD2P1 knockdown were analyzed by CCK8 and flow cytometry for the proliferation and cycling of BCa cells. Figure 3A shows the overexpression treatment of GTF2IRD2P1 that significantly promoted GTF2IRD2P1 expression compared to the vector (*p* < 0.0001). Treatments that knocked down both targets of GTF2IRD2P1 significantly inhibited GTF2IRD2P1 expression compared to the negative control (NC) (*p* < 0.01). In addition, CCK8 experiments showed that GTF2IRD2P1 OE inhibited the proliferation of BCa cells. However, GTF2IRD2P1 knockdown promoted BCa cell proliferation (Fig. 3B). Flow cytometry analysis also showed that GTF2IRD2P1 OE significantly enhanced cell cycle arrest, and GTF2IRD2P1 knockdown attenuated cell cycle arrest (Fig. 3C). We examined the protein levels of key components in the cell cycle after GTF2IRD2P1 OE and GTF2IRD2P1 knockdown to further reveal the critical role of GTF2IRD2P1 in the cell cycle. Western blot analysis showed that GTF2IRD2P1 overexpression downregulated Cyclin A2 and Cyclin D1 protein levels in UM-UC-3 cells. However, the knockdown of GTF2IRD2P1 upregulated the protein levels of Cyclin A2 and Cyclin D1 in T24 cells (Fig. 3D). Taken together, the above results suggests that changes in GTF2IRD2P1 expression cause corresponding changes in cell proliferation and cell cycle in *in vitro* experiments.

### Effect and role of GTF2IRD2P1 in cell proliferation and cell cycle by *in vivo* experiments

**Figure 4:**
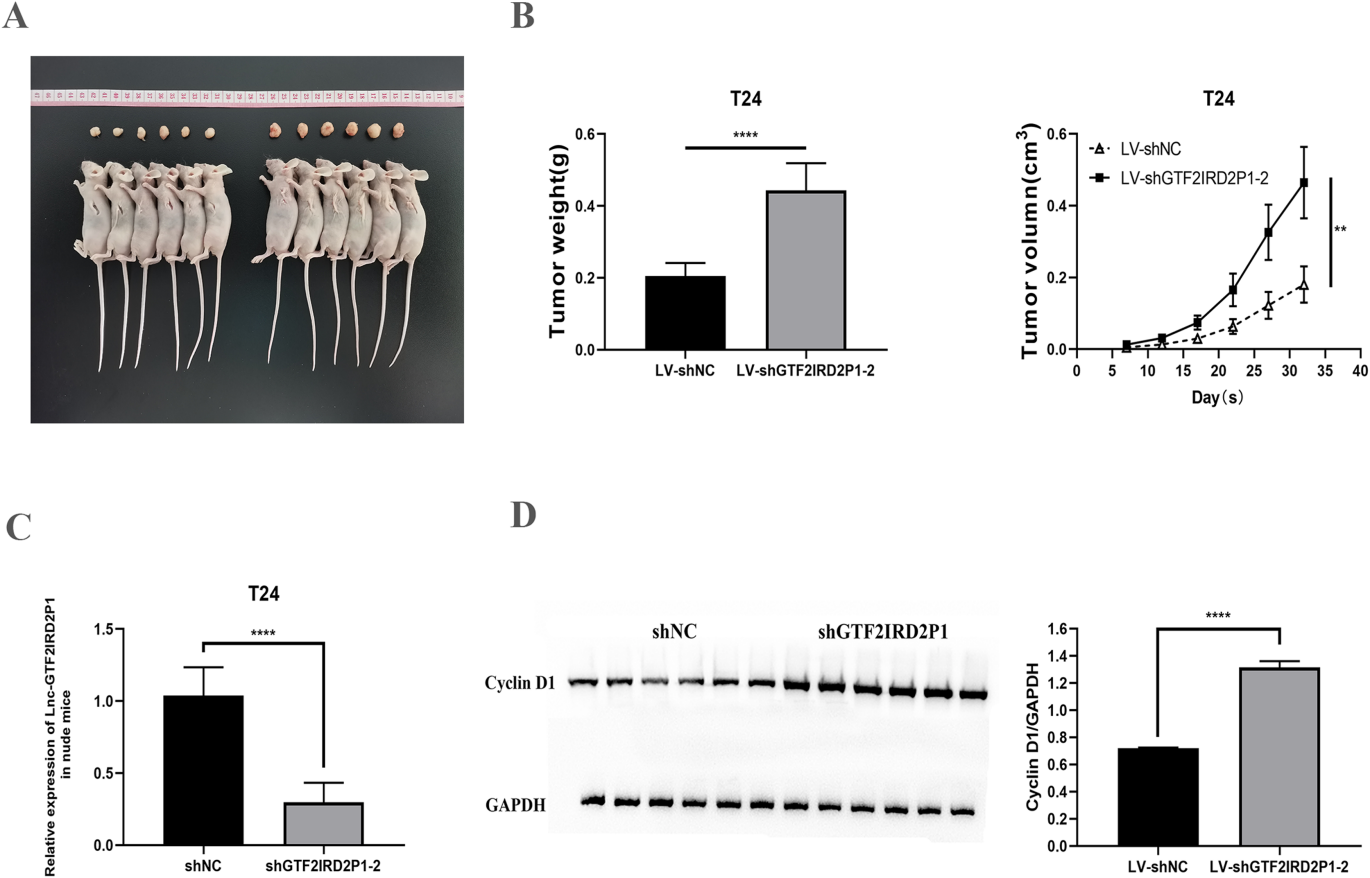
Effect of GTF2IRD2P1 on proliferation and cell cycle of BCa cells by *in vivo* assay. (A and B) Solid tumors taken from subcutaneous transplanted tumor tissue of mice. (C) qRT-PCR to detect the expression of GTF2IRD2P1 in nude mouse tumors. (D) Western blot analysis of cell cycle protein expression in nude mouse tumors. p** < 0.01, p**** < 0.0001. BCa, bladder cancer.

We confirmed the regulation of BCa development *in vivo* by GTF2IRD2P1 using a T24 cell nude mouse xenograft model. The LV-NC group and the LV-shGTF2IRD2P1-2 group were inoculated with T24 cells, respectively. The tumors were dissected as shown in Fig. 4A. In terms of tumor phenotype, LV-shGTF2IRD2P1-2 promoted tumor growth, and GTF2IRD2P1 knockdown significantly increased the weight and volume of the tumor (Fig. 4B). Meanwhile, the differential expression levels of GTF2IRD2P1 in nude mouse tumors were detected by qRT-PCR (Fig. 4C). The level of cyclin D1, a cell cycle protein, was analyzed by Western blot in nude mouse tumors (Fig. 4D). Thus, these data suggest that the low expression of GTF2IRD2P1 promoted cell proliferation and cell cycle protein expression in *in vivo* experiments.

### GTF2IRD2P1 negatively regulates the Wnt signaling pathway

**Figure 5:**
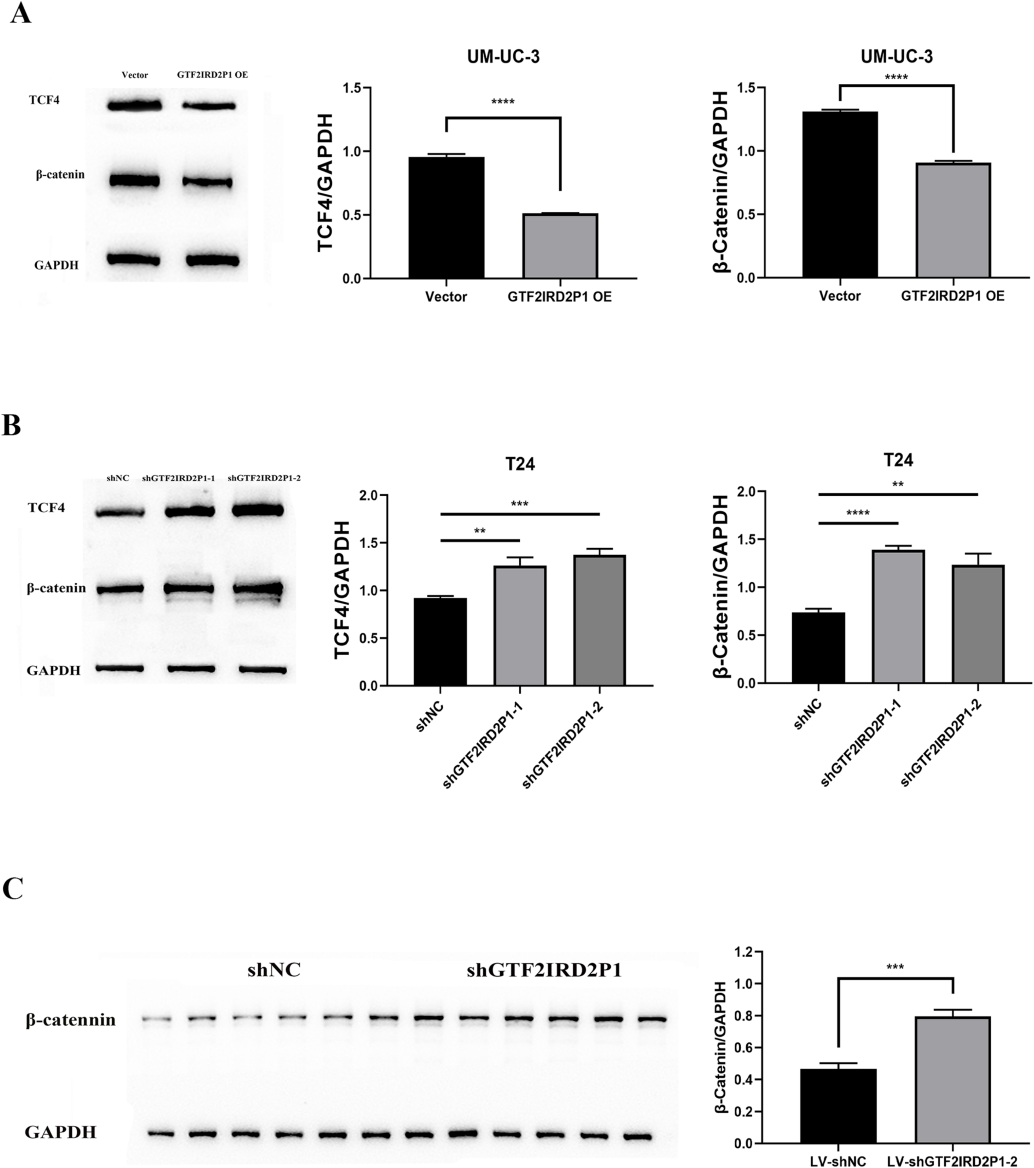
The role of GTF2IRD2P1 in the regulation of BCa in the Wnt signaling pathway. (A) Western blot to detect the Wnt signaling pathway key protein expression in UM-UC-3 cells. (B) Western blot to detect the Wnt signaling pathway key protein expression in T24 cells. (C) Western blot to detect the Wnt signaling pathway key protein expression in nude mouse tumors. p** < 0.01, p*** < 0.001, p**** < 0.0001. BCa, bladder cancer.

The critical role of GTF2IRD2P1 in the Wnt signaling pathway for BCa progression was demonstrated. We examined the expression levels of key protein molecules in the Wnt signaling pathway following GTF2IRD2P1 overexpression and GTF2IRD2P1 silencing in UM-UC-3 and T24 BCa cell lines, respectively, and we also validated it in *in vivo* animal experiments. Western blotting analysis showed that GTF2IRD2P1 overexpression downregulated β-catenin and TCF4 levels in UM-UC-3 cells (Fig. 5A). In contrast, GTF2IRD2P1 knockdown activated Wnt signaling in T24 cells (Fig. 5B). The same result was seen in the nude mouse model (Fig. 5C). Therefore, GTF2IRD2P1 may mediate Wnt signaling in BCa cells in both *in vitro* and *in vivo* experiments.

### Rescue assay: pathway inhibitor can suppress the pro-carcinogenic effect of GTF2IRD2P1

**Figure 6:**
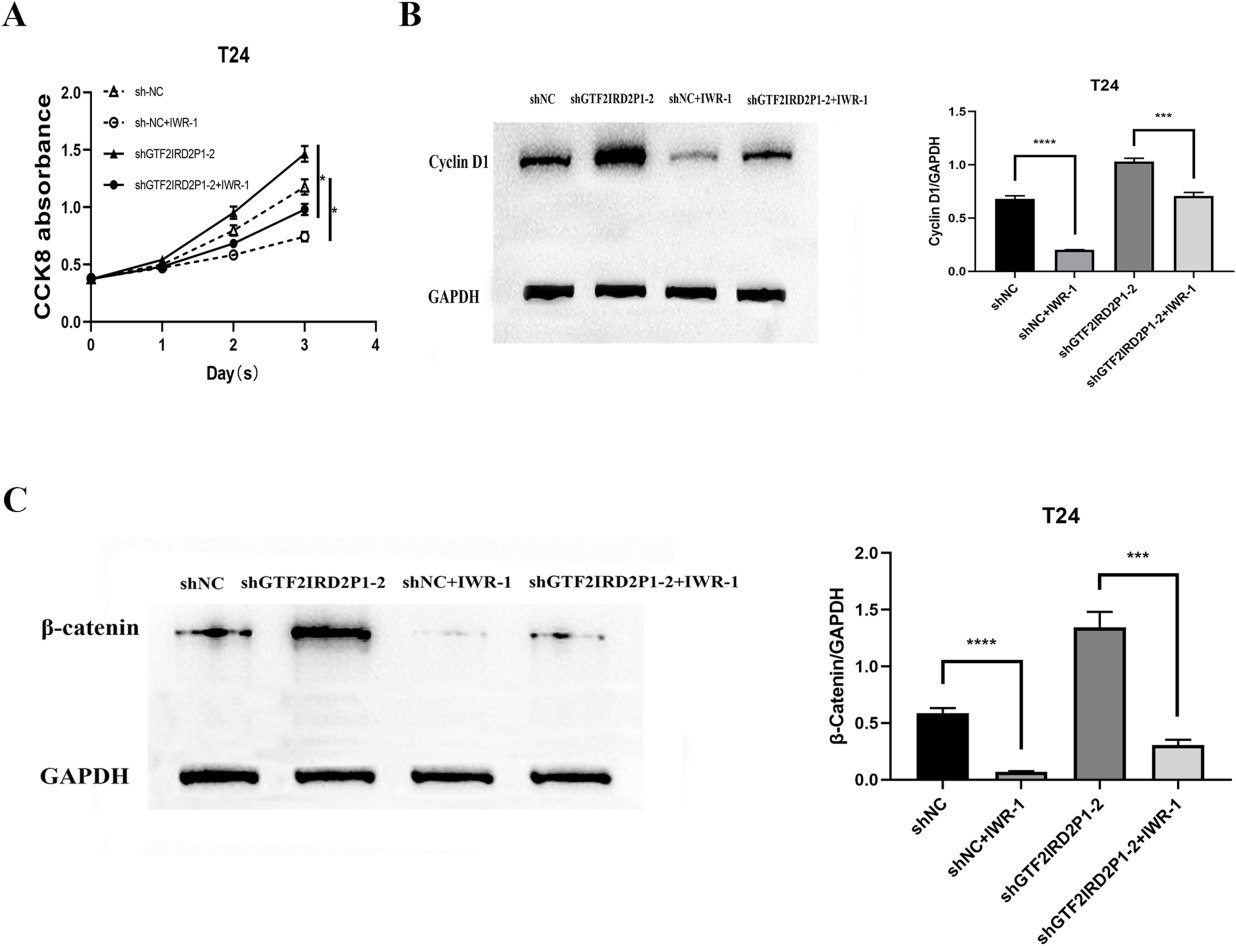
Rescue assay: Wnt signaling pathway inhibitor IWR-1 rescues the pro-carcinogenic effect of GTF2IRD2P1 in BCa. (A) CCK8 assay to detect the effect of IWR-1 on the proliferation of GTF2IRD2P1 in BCa cells. (B) Western blot to detect the role of IWR-1 on the expression of cyclins in GTF2IRD2P1 in BCa cells. (C) Western blot to detect the role of IWR-1 on the expression of key proteins of the Wnt signaling pathway in GTF2IRD2P1 in BCa cells. p* < 0.05, p*** < 0.001, p**** < 0.0001. BCa, bladder cancer.

Finally, IWR-1, a Wnt signaling pathway inhibitor, was used to validate the function of GTF2IRD2P1 in the evolution of BCa. We chose to add IWR-1 as a negative control and knockdown of GTF2IRD2P1 groups in T24 cells, respectively. This resulted in the groups of shNC, shNC+IWR-1, shGTF2IRD2P1, and shGTF2IRD2P1+IWR-1. CCK8 experiments showed that IWR-1 could significantly rescue the knockdown of GTF2IRD2P1 on BCa cells (Fig. 6A). Western blot analysis showed that IWR-1 not only inhibited the upregulation of cyclin D1 by GTF2IRD2P1 knockdown (Fig. 6B) but also attenuated the promotion of the key protein β-catenin in the Wnt signaling pathway by the knockout of GTF2IRD2P1 (Fig. 6C). Therefore, the low expression of GTF2IRD2P1 may contribute to the growth of urinary BCa cells by activating the Wnt signaling pathway.

## Discussion

The unrestricted proliferation of cells is one of the greatest difficulties to overcome for successful tumor treatment. It is also one of the main reasons for the low survival rate of BCa (Koivisto et al., 2019). Therefore, there is an urgent need to find suitable therapeutic targets to reduce the high mortality rate of BCa patients. There has been growing evidence in the past decade that lncRNAs play a major role in the mechanisms of pathogenesis for various carcinomas, including BCa (Li et al., 2020). We believe that GTF2IRD2P1 is a prospective target for BCa treatment. To the best of our knowledge, the involvement of lncRNA-GTF2IRD2P1 in the progression and metastasis of oral squamous carcinoma was first reported in 2015 (Zhang et al., 2015), but its role in BCa is not clear. We studied BCa and paracancerous tissues from 30 total BCa resection patients and found that GTF2IRD2P1 showed low expression in BCa tissues and differed significantly from its expression in paracancerous tissues. Furthermore, GTF2IRD2P1 knockdown significantly promoted the malignant phenotype of cell proliferation, and the overexpression of GTF2IRD2P1 suppressed the aforementioned malignant phenotype. Mechanistically, we found that cell cycle proteins Cyclin A2 and Cyclin D1 were up-regulated after GTF2IRD2P1 knockdown, as were Wnt signaling pathway proteins β-catenin and TCF4. However, the overexpression of GTF2IRD2P1 had an opposite effect. Interestingly, the Wnt signaling pathway inhibitor IWR-1 was shown to reverse the effect of GTF2IRD2P1 knockdown on the propagation of BCa cells. Furthermore, xenograft studies showed that GTF2IRD2P1 knockdown significantly promoted neoplastic growth *in vivo*. These results indicate that GTF2IRD2P1 may be a promising biomarker for the diagnosis and prognosis of BCa and may provide a new therapeutic target for the treatment of BCa.

The signaling pathway of Wnt crosstalk leads to a variety of celluslar activities, including embryogenesis, wound healing, stem cell stability, the homeostasis of calcium and tissues, and the growth of cells (Mohammed et al., 2016). The development of mutations and epigenetic alterations in key molecules of the Wnt/β-catenin classical pathway is strongly associated with oncogenesis, progression of drug resistance, and improvements in survival. Therefore Wnt signaling components are considered to be essential indicators for the diagnosis and prognosis of cancer and may provide new therapeutic targets (Garg & Maurya, 2019). Wnt/β-catenin signaling has been widely reported to play an important role in the malignant progression of BCa (Mao et al., 2018; Yuan et al., 2017). For example, the high expression of Linc00152 promotes BCa hyperproliferation and migration mediated by the activation of the Wnt/β-catenin signaling pathway (Xian-li et al., 2019). The long non-coding RNA SNHG20 plays a role in promoting cancer in the bladder by activating the Wnt/β-catenin signaling pathway (Zhao et al., 2018). However, we have shown that GTF2IRD2P1 can inhibit the Wnt/β-catenin signaling in BCa cells and that GTF2IRD2P1 exerts its effect on carcinogenesis in BCa by inactivating the signaling pathway of Wnt/β-catenin.

There are some limitations of our work. First, there was a small clinical specimen volume, which should be increased in future studies. Secondly, although we demonstrated that the mechanism of GTF2IRD2P1 negatively regulated the Wnt signaling pathway, it’s specific role in BCa is still unclear. Therefore, its role should be further explored in future studies.

## Conclusions

In summary, GTF2IRD2P1 inhibits BCa development by regulating Wnt signaling. Notably, the downregulation of GTF2IRD2P1 promotes the progression of BCa *via* the activation of Wnt signaling. Our findings from this study suggest that GTF2IRD2P1 may be a prospective treatment target and a biomarker of prognosis for BCa patients.

## Supplemental Information

10.7717/peerj.13220/supp-1 Supplemental Information 1 Raw Data: results. The result of bioinformatics analysis; the result of qPCR detection; the result of Western Blot; the result of CCK8 experiment; result of animal experiment.

10.7717/peerj.13220/supp-2 Supplemental Information 2 Raw Data: RT-qPCR results for all clinical samples.

10.7717/peerj.13220/supp-3 Supplemental Information 3 Uncropped Blots. A Western blot; an animal experiment picture; a cell cycle picture.

10.7717/peerj.13220/supp-4 Supplemental Information 4 Uncropped Blots: β-catenin (Five clinical specimens).

10.7717/peerj.13220/supp-5 Supplemental Information 5 Uncropped Blots: Expression levels of β-catenin protein in five patients with bladder cancer.

## Additional Information and Declarations

### Data Availability

The following information was supplied regarding data availability: The raw measurements, figures and images are available in the Supplemental Files.
